# A Depressive Endophenotype of Mild Cognitive Impairment and Alzheimer’s Disease

**DOI:** 10.1371/journal.pone.0068848

**Published:** 2013-07-11

**Authors:** Leigh A. Johnson, James R. Hall, Sid E. O’Bryant

**Affiliations:** 1 Department of Internal Medicine, University of North Texas Health Science Center, Fort Worth, Texas, United States of America; 2 Institute for Aging & Alzheimer’s Disease Research, University of North Texas Health Science Center, Fort Worth, Texas, United States of America; 3 Department of Psychiatry, University of North Texas Health Science Center, Fort Worth, Texas, United States of America; University Of São Paulo, Brazil

## Abstract

**Background:**

Alzheimer’s disease (AD) is a devastating public health problem that affects over 5.4 million Americans. Depression increases the risk of Mild Cognitive Impairment (MCI) and AD. By understanding the influence of depression on cognition, the potential exists to identify subgroups of depressed elders at greater risk for cognitive decline and AD. The current study sought to: 1) clinically identify a sub group of geriatric patients who suffer from depression related cognitive impairment; 2) cross validate this depressive endophenotype of MCI/AD in an independent cohort.

**Methods and Findings:**

Data was analyzed from 519 participants of Project FRONTIER. Depression was assessed with the GDS30 and cognition was assessed using the EXIT 25 and RBANS. Five GDS items were used to create the Depressive endophenotype of MCI and AD (DepE). DepE was significantly negatively related to RBANS index scores of Immediate Memory (B=-2.22, SE=.37, p<0.001), visuospatial skills (B=-1.11, SE=0.26, p<0.001), Language (B=-1.03, SE=0.21, p<0.001), Attention (B=-2.56, SE=0.49, p<0.001), and Delayed Memory (B=-1.54, SE = 037, p<0.001), and higher DepE scores were related to poorer executive functioning (EXIT25; B=0.65, SE=0.19, p=0.001). DepE scores significantly increased risk for MCI diagnosis (odds ratio [OR] = 2.04; 95% CI=1.54-2.69). Data from 235 participants in the TARCC (Texas Alzheimer’s Research & Care Consortium) were analyzed for cross-validation of findings in an independent cohort. The DepE was significantly related to poorer scores on all measures, and a significantly predicted of cognitive change over 12- and 24-months.

**Conclusion:**

The current findings suggest that a depressive endophenotype of MCI and AD exists and can be clinically identified using the GDS-30. Higher scores increased risk for MCI and was cross-validated by predicting AD in the TARCC. A key purpose for the search for distinct subgroups of individuals at risk for AD and MCI is to identify novel treatment and preventative opportunities.

## Introduction

Alzheimer’s disease (AD) is the most common form of neurodegenerative dementia with over 5.4 million Americans suffering from the disease [1]. This number is expected to reach 7.7 million in 2030, resulting in more than a 50% increase from the current prevalence rates [2]. Every 71 seconds an American develops AD and by mid-century, it is expected that this timeframe will accelerate to one new case every 33 seconds [2]. AD is the 7^th^ leading cause of death in the U.S. and the 5^th^ leading cause of death for those over 65 [2]. AD poses a tremendous public health problem in terms of care, lost wages, and caregiver burden. Mild Cognitive Impairment (MCI) is a prodromal category to AD with an annual conversion rate from MCI to AD of 10-15%. It is estimated that between 10–30% of all adults age 65 and above suffer from MCI [3]. When combined with the 13% prevalence rate of AD among elders [3], anywhere between 8 and 14 million of Americans age 65 and above currently suffer from MCI or AD. Given the population aging trends, these numbers will grow drastically in the near future. Despite the looming healthcare crisis that is MCI/AD, to date there are no effective strategies for preventing or halting progression of the disease. It is our view that the key to successfully addressing MCI/AD lies in the very complexity of the disease itself. We hypothesize that MCI and AD can be deconstructed into multiple subgroups (or endophenotypes), each of which may offer novel opportunities for treatment and/or prevention.

The term endophenotype [4] has been discussed frequently in psychiatry and provides a way for identifying subgroups of clinical phenotypes [5]. In the recent literature, researchers have begun searching for endophenotypes of AD as a method deconstructing the disease for targeting sub-groups that may respond differentially to treatments and explain prior treatment failures. In our work, we have proposed an inflammatory endophenotype of AD [6,7] as well as a neurotrophic factor endophenotype of AD [8] based on blood-based biomarkers. Other endophenotypes have now been proposed based on neuropathology [9], neuroimaging [10,11], genetics [12], and cerebrospinal fluid markers [13]. Neuropsychiatric endophenotypes of AD have also been proposed, which included depression [14] and is consistent with our data. The identification of MCI/AD endophenotypes has the potential to provide a paradigm shift in how the disease is approached in terms of treatment and prevention studies from a personalized medicine standpoint. The identification of endophenotypes would also support novel approaches to a mechanistic understanding of MCI/AD as well as cognitive dysfunction/decline more broadly.

Depression has been shown to be a risk factor for as well as prodromal symptom of AD [15]. Depression is the most frequently reported psychiatric symptom among patients with MCI [16], with one-fifth reporting moderate-to-high levels of depression [17] and depression increases risk for progression from MCI to AD [18]. Increasing levels of depression have been found to be significantly linked to poorer neuropsychological scores on tests of executive functioning, psychomotor speed, motor functioning, and memory [19–22] with comorbid depression and cognitive dysfunction being associated with greater impairment in activities of daily living as well as decreased quality of life [23–25]. However, this work has focused on global depressive diagnoses or screening instrument scores without any prior work explicitly identifying the specific subset of depressive symptoms that increase risk for MCI or AD. It is our hypothesis that there exists a depressive symptom pattern that is explicitly associated with cognitive impairment independent of global depressive symptoms. This work is designed to identify which patients suffering from depression are at greatest risk for cognitive dysfunction and potentially which patients suffering from cognitive dysfunction may benefit cognitively from anti-depressant therapies (pharmaceutical and/or psychotherapeutic).

The current study was undertaken to (1) identify the specific depressive symptom pattern that conveys risk for MCI and AD, independent of global depression scores, and (2) cross-validate the findings in an independent cohort.

## Methods

This research was conducted under IRB approved protocols with each participant (and/or informants for cognitively impaired persons) providing written informed consent. Project FRONTIER is conducted under the approval of Texas Tech Health Sciences Center IRB Board, L06-028. This archival analysis was conducted from a de-identified database under the approval of the University of North Texas Health Sciences Center IRB Review Board, protocol number 2012-071. The TARC is conducted under University of North Texas Health Sciences Center IRB protocol #2007-137.

### Participants

Data were analyzed from two ongoing studies of cognitive aging, Project FRONTIER and TARCC.

#### Project FRONTIER (Facing Rural Obstacles Now to health Through Intervention, Education and Research)

Data was analyzed from 519 participants of Project FRONTIER, which is an ongoing epidemiological study of factors impacting rural aging and health. Average age and education of the total sample was 61.49 (sd =12.7) and 10.53 (sd =8.9), respectively. A total of 235 participants self-identified as Mexican Americans. Descriptive statistics of the sample, by training versus validation sample, can be found in Table 1. Project FRONTIER [26–31] utilizes a community-based participatory research (CBPR) approach to solicit anyone age 40 and above locating in three counties on the Texas–New Mexico border (Cochran, Bailey, and Parmer Counties). CBPR involves partnering communities with scientific groups to conduct studies of human disease that is growing rapidly in terms of use and acceptance in the scientific community. CBPR is particularly useful when working with underserved communities that may not respond to classic approaches (e.g., random digit dialing, mail surveys); CBPR is supported by and recommended for rural research by the National Institute of Environmental Health Sciences [32] and we have demonstrated that the sample recruited closely resembles the total eligible population of these rural communities [28]. We partnered with the local hospitals and clinics as well as the senior citizen’s organizations. Community recruiters and research staff presented information about the study at community events, churches, food banks, and local businesses.

**Table 1:** Demographic characteristics of samples.

	**FRONTIER Training Sample N=256**	**FRONTIER Test Sample N=263**	**TARCC Baseline N=235**	**TARCC 12-mo Follow-up N=142**	**TARCC 24-mo Follow-up N=90**
Age (yrs)	61.07 (12.4)	61.5 (13.0)	73.5 (9.8)	72.9 (9.7)	74.3 (10.0)
Range	40-94	40-94	55-96	55-94	56-94
Gender (% male)	31	30	31	31	31
Education (yrs)	10.78 (4.2)	10.92 (4.3)	14.37 (3.1)	14.53 (3.2)	14.82 (3.0)
Range	0-20	0-20	3-22	3-22	6-20
GDS total score	10.47 (5.0)	10.86 (5.1)	4.63 (4.2)	3.79 (4.3)	4.02 (3.7)
Range	0-26	0-26	0-23	0-30	0-20
GDS-DepE^1^	1.00 (1.3)	1.00 (1.4)	0.76 (1.0)	0.52 (0.8)	0.61 (0.90)
Range	0-5	0-5	0-4	0-4	0-4
MMSE	27.6 (2.3)	28.0 (2.6)	24.1 (6.6)	24.6 (6.3)	23.7 (6.5)
RBANS Imm Mem	93.3 (17.5)	93.3 (18.4)	−	−	−
RBANS Visuo	81.3 (15.4)	82.0 (17.1)	−	−	−
RBANS Lang	93.0 (12.0)	93.3 (13.0)	−	−	−
RBANS Attention	87.3 (20.5)	88.5 (22.3)	−	−	−
RBANS Del Mem	92.3 (15.3)	93.0 (16.0)	−	−	−
EXIT25	7.2 (4.6)	6.9 (4.7)	−	−	−
DepE Frequency^1^					
0	54%	55%	55%	65%	60%
1	20%	20%	22%	22%	25%
2	9%	10%	16%	11%	11%
3	9%	8%	5%	1%	3%
4	6%	4%	2%	1%	1%
5	2%	3%	0%	0%	0%

1 GDS-DepE= GDS Depression Endophenotype.

#### TARCC (Texas Alzheimer’s Research & Care Consortium)

Data from 235 participants (NC=117, MCI n=10, AD n=108) were analyzed for cross-validation of findings in an independent cohort. Participants completed a standardized examination at one of the five participating site (Texas Tech University Health Sciences Center, University of North Texas Health Science Center, University of Texas Southwestern Medical Center, University of Texas Health Science Center – San Antonio and Baylor College of Medicine) dementia specialty clinics. Inclusion criteria for TARCC are age 50 or above with diagnosis of Probable AD [33], MCI [34] or normal control (NC) [35], Mini Mental State Exam (MMSE) score > 11 (at entry), and available informant. Participants with a Hachinski Ischemic Score > than four, history of stroke or have current cancer, neurological disease (e.g. Parkinson’s disease), acute inflammatory disorders (multiple sclerosis, rheumatoid arthritis) or urinary current infections are excluded. Additionally, participants with a significant psychiatric history and severe depression at the time of initial assessment are excluded from the study. Data from both of these studies have been published extensively elsewhere [7,27,28,30,31,36–40].

### Procedures

Each study utilizes a detailed protocol that includes a medical exam, interview (with participant and a reliable informant), and neuropsychological testing. Diagnoses were assigned by consensus review according to published criteria for AD [41], MCI [34] and NC [35].

### Measures

In both cohorts, the GDS [42] was administered, which is a thirty-item yes/no scale originally developed to measure depressive symptoms among samples of older adults. Scores (yes/no) range from 0-30 with higher scores representing greater symptom severity. The GDS has been shown to have adequate psychometric properties across a wide range of age groups [42]. Data from the Repeatable Battery for the Assessment of Neuropsychological Status (RBANS) [43] and the Exit Interview (EXIT25) [44] were analyzed from Project FRONTIER. The RBANS is a brief neuropsychological instrument that assesses immediate and delayed memory, visuospatial skills as well as attention and language abilities. It encompasses a total of 12 subtests that combine to create five indices. The RBANS has accumulated a large amount of normative data [43] and has good psychometric properties and diagnostic accuracy [45,46]. The EXIT25 [44] is a well-validated global measure of executive control that covers a range of tasks including sequencing, fluency, anomalous sentence repetition, thematic perception, automatic behaviors, go-no-go, automatic behavior, as well as others. EXIT25 scores are significantly related with other validated measures of executive functioning such as the Mini Mental Status Exam, the Wisconsin Card Sorting Task, Trail Making Test A and B, and the Serial Attention Test [47]. Scores range from zero to 50 with higher scores suggestive of greater impairment; a cutoff point of 15 out of 50 best discriminates non-demented elderly controls from both cortical and non-cortical dementing illness [44]. The TARCC neuropsychology core battery consists of neuropsychological instruments administered as part of the established Alzheimer’s disease clinical/research platform at each participating institution. For the purposes of the current study, scores on the following tests were analyzed: WMS Logical Memory & Visual Reproduction, Boston Naming Test, verbal fluency (FAS), Mini-Mental State Examination (MMSE) [48], and the Clinical Dementia Rating scale (CDR) [49]. In order to equate scores all raw scores were converted to scale scores based on previously published normative data [50]. For the Boston Naming Test, the current group recently conducted an independent study that demonstrated the psychometric properties of an estimated 60-item BNT score that can be calculated from 30-item versions [51]. Adjusted scale scores were utilized as dependent variables in analyses.

### Statistical Analyses

Analyses of demographic characteristics between ethnic groups were conducted via ANOVA (continuous) or χ^2^ (categorical) analyses. The development and validation of the DepE utilized X^2^ and logistic and linear regression.

#### Development and Validation

The development of the Depressive Endophenotype(DepE) of MCI and AD. The identification of the specific depressive symptoms related to cognitive impairment (i.e. the depressive endophenotype of cognitive impairment [DepE] was completed via aseries of steps. Step 1-one-half of the sample from Project FRONTIER was randomly assigned to a training sample with the remainder of the cohort assigned to the test sample. Step 2-, differential endorsement of each item of the GDS by MCI versus normal diagnostic category was undertaken via χ^2^. Any items that were endorsed significantly differently between the two groups at p<0.05 were retained for the DepE. Step3-the DepE was applied to the test sample from Project FRONTIER to determine its relation to cognitive functioning and MCI diagnosis.

The link between DepE and neuropsychological functioning was carried out via linear regression with the DepE entered as predictor variables with neuropsychological test scores as the outcome variables; age, gender, education, ethnicity and ApoEε4 genotype were entered as covariates. Logistic regression was undertaken to examine the risk of MCI diagnosis as a function of increasing items endorsed on the DepE; age, gender, education, ethnicity and ApoEε4 genotype were entered as covariates. Cross-Validation. In order to cross-validate the DepE in an independent cohort, data from the TARCC was analyzed. Item-level GDS scores were available for 235 participants at baseline (NC=117, MCI n=10, AD n=108), 138 participants (AD n=67, 1 MCI, NC n=70) had requisite 12-month follow-up data, and 64 participants (AD n=35, NC n=29) had 24-month follow-up. The DepE was applied directly to the TARCC cohort without modification. The link between the DepE index and baseline neuropsychological test scores was carried out via linear regression. The relative risk of being diagnosed with AD associated with DepE was carried out via logistic regression. Due to the sample size of item-level GDS scores available, separate analyses for MCI only could not be carried out.

## Results

### Development and Validation

Demographic characteristics of the training and test samples from Project FRONTIER can be found in Table 1. In the training sample, there were 204 participants consensus judged as cognitively normal and 52 as MCI. In the training sample, the following items were significantly endorsed more often among the MCI group than the normal cognition group: feeling of worse memory problems (χ^2^=12.39, p<0.001), feeling downhearted and blue (χ^2^=6.97, p=0.008), feeling worthless (χ^2^=5.58, p=0.02), frequently feel like crying (χ^2^=6.50, p=0.01), and trouble concentrating (χ^2^=7.82, p=0.005). Of note, a positive endorsement on each of these items is in the direction of positive depression, therefore reverse scoring was not needed. The DepE was generated by summing the responses of each person on these 5 items resulting in a score ranging from 0 to 5. The mean DepE score in the training sample was 1.0 (sd = 1.3, range = 0-5).

The test sample consisted of 203 participants designated as cognitively normal on consensus review and 60 MCI. In the test sample, the mean DepE score was 1.0 (sd = 1.4, range= 0.5) with MCI cases scoring significantly higher (1.7, sd = 1.8) than cognitively normal participants (0.8, sd = 1.1) (t=3.59, p=0.001). The DepE was significantly negatively related to RBANS index scores of Immediate Memory (B=-2.22, SE=.37, p<0.001), Visuospatial skills (B=-1.11, SE=0.26, p<0.001), Language (B=-1.03, SE=0.21, p<0.001), Attention (B=-2.56, SE=0.49, p<0.001), and Delayed Memory (B=-1.54, SE = 037, p<0.001). Higher DepE scores were also related to significantly poorer executive functioning (EXIT25; B=0.65, SE=0.19, p=0.001). To illustrate the independent impact of DepE scores on neuropsychological testing, analyses were re-run including GDS total scores (minus DepE items). DepE scores were significantly related to RBANS indices of Immediate Memory, Visuospatial skills, Language, Attention, and Delayed Memory independent of GDS total scores. GDS total scores were only significantly related to Delayed Memory and Attention scores. GDS total scores were significantly related to EXIT scores, but not DepE scores. Therefore, DepE scores appear to be most related to memory abilities with global depression scores related to measures of attention and executive functioning. Table 2 shows the correlations between DepE scores and demographic and neuropsychological data in the FRONTIER test sample.

**Table 2:** Correlation of DepE with demographic variables and cognitive test scores.

	**R^2^**	**p-value**
**FRONTIER**		
Age – Training Sample	-0.08	0.21
Age – Test Sample	-0.20	=0.001
Education – Training Sample	-0.11	=0.06
Education – Test Sample	-0.13	=0.03
Gender – Training Sample	-0.03	095.
Gender – Test Sample	0.07	0.27
MMSE – Training Sample	-0.31	<0.001
MMSE – Test Sample	-0.25	<0.001
RBANS Imm Mem – Training Sample^1,2^	-0.29	<0.001
RBANS Imm Mem – Test Sample^1,2^	-0.26	<0.001
RBANS Visuospatial – Training Sample^1,3^	-0.17	<0.001
RBANS Visuospatial – Test Sample^1,3^	-0.22	<0.001
RBANS Language – Training Sample^1^	-0.24	<0.001
RBANS Language – Test Sample^1^	-0.24	<0.001
MMSE – Training Sample	-0.31	<0.001
MMSE – Test Sample	-0.25	<0.001
RBANS Attention – Training Sample^1^	-0.22	<0.001
RBANS Attention – Test Sample^1^	-0.17	0.005
RBANS Del Mem – Training Sample^1,4^	-0.21	0.001
RBANS Del Mem – Test Sample^1,4^	-0.20	0.001
EXIT-Training Sample	0.19	0.003
EXIT-Test Sample	0.16	0.01
**TARCC**		
Age	0.15	0.03
Education	-0.21	0.002
Gender	0.01	0.83
MMSE	-0.25	<0.001
FAS	-0.28	<0.001
BNT	-0.25	<0.001
Logical Mem I	-0.41	<0.001
Logical Mem II	-0.44	<0.001
Visual Repro I	-0.47	<0.001
Visual Repro II	-0.56	<0.001

1 RBANS scores are raw index scores.

2 Imm Mem = Immediate Memory Index raw score.

3 Visuospatial = Visuospatial Index raw score.

4 Del Mem = Delayed Memory raw index score.

Next, logistic regression was utilized to determine the risk of being diagnosed with MCI as a function of DepE scores within the test sample. DepE scores significantly increased risk for MCI diagnosis (odds ratio [OR] = 2.04; 95% CI=1.54-2.69), which was the only significant predictor aside from age (OR=1.09; 95% CI=1.05-1.13) and education (OR=0.82; 95% CI=0.71-0.95). In a conditional stepwise forward logistic regression, age entered into the model first, followed by the DepE and then education; no other variables entered into the model.

### Cross-Validation

Mean DepE scores were significantly higher among AD cases (1.39, SD=1.27, range = 0-5) as compared to controls (0.37, SD=0.71, range 0-3) (F=27.88, p<0.001). MCI patients scored between AD cases and normal controls (0.90, SD=1.20, range = 0-3) though the sample size was too small for statistical comparison. Based on the findings from Project FRONTIER, neuropsychological tests of memory and language were evaluated in TARCC as were scores of global cognition and disease severity. Correlations between DepE scores and demographic and neuropsychological data are presented in Table 2.

The results of the linear analyses indicated that DepE scores were significantly related to poorer scores on all measures (see Table 3). To illustrate the specific DepE – cognition link independent of global depression scores, these baseline analyses were re-run including GDS total score (minus DepE questions) in the models. With the exception of FAS, results remained unchanged with DepE scores being a significant predictor of cognitive test scores with total GDS scores not being significantly related to any cognitive outcome variables. For FAS, total GDS score was significantly related to test scores (p=0.03) with DepE scores not being significant (p=0.07).

**Table 3:** Relation between DepE & DepE2 scores and neuropsychological test scores in TARCC.

	**Mean (SD)**	**B(SE)**	**t-value**	**p-value**
LMI	8.4 (4.9)	-1.49 (0.29)	-5.13	<0.001
LMII	8.7 (5.2)	-1.62 (0.29)	-5.67	<0.001
VRI	8.7 (4.5)	-1.40 (0.31)	-4.58	<0.001
VRII	9.5 (4.9)	-1.79 (0.30)	-6.04	<0.001
BNT	8.7 (4.5)	-0.82 (0.23)	-3.65	<0.001
FAS	8.9 (3.7)	-0.96 (0.21)	-3.56	<0.001
MMSE	24.1 (6.6)	-0.96 (0.30)	-3.17	0.002
CDR SB	3.9 (4.9)	0.80 (0.23)	3.60	<0.001
**DepE2 – T2 Neuropsychological scores**				
LMI	10.7 (5.3)	-0.86 (0.43)		0.04
LMII	11.0 (5.4)	-1.04 (0.43)	-2.44	0.01
VRI	9.5 (5.1)	-1.04 (0.49)	-2.75	0.007
VRII	11.3 (5.0)	-1.27 (0.48)	-2.62	0.01
BNT	9.1 (4.4)	-1.33 (0.41)	-3.21	0.002
FAS	9.9 (3.9)	-0.85 (0.33)	-2.60	0.01
MMSE	24.6 (6.3)	-1.28 (0.52)	-2.48	0.01
CDR SB	4.3 (5.4)	0.84 (0.23)	3.60	<0.001
**DepE -baseline and 24 month change scores**				
ΔMMSE	1.9 (3.5)	1.17 (0.36)	3.27	0.002
ΔCDR-SB	-2.2 (3.0)	-0.80 (0.33)	-2.41	0.02

Next a logistic regression model was created with AD versus normal control as the outcome variable; age, gender, ethnicity, education, ApoEε4 presence (yes/no), GDS total score and DepE scores were entered as the predictor variables. Age (OR=1.18, 95% CI= 1.12-1.24, p<0.001), ApoEε4 status (OR=2.42, 95% CI=1.13-5.19, p=0.02) and DepE scores (OR=2.49, 95% CI=1.40-4.43, p=0.002) were the only significant predictors of AD status. In the forward conditional stepwise logistic regression, the order of entry into the model was age, DepE, and ApoEε4 status. The DepE alone was a significant predictor of AD status using receiver operating characteristic (ROC) curve analysis (Area Under the Curve [AUC] = 0.74 (95% CI=0.68-0.81), p<0.001). The results remained consistent when predicting AD/MCI status. However, even though DepE index scores were significantly related to AD status in both ApoEε4 carriers (OR=2.81, 95% CI=1.2-6.5, p=0.02) and ApoEε4 non-carriers (OR=8.03, 95% CI=3.06-21.08, p<0.001), the effect was clearly stronger for non-carriers.

The TARCC is a longitudinal cohort and, therefore, preliminary analyses were conducted with data from participants who had available DepE scores at 12- and 24-month follow-up evaluations (DepE2, DepE3). A total of 142 participants (AD n=67, 1 MCI, NC n=70, 4 other) had available information for 12-month analyses. The mean (standard deviation) age, education and DepE2 scores for the participants (44 men and 98 women) was as follows: 72.9 (9.7), 14.5 (3.2), and 0.6 (0.9), respectively. DepE2 scores were significantly associated with poorer scores on all measures (see Table 2). DepE2 was also a significant predictor of AD status (compared to controls) at 12-month follow-up visits: OR=4.02 (95% CI=1.73-9.32, p=0.001). A total of 90 participants (AD n=41, NC n=49) had 24-month follow-up data available for calculation of a DepE, which continued to be a significant predictor of AD case status (OR = 3.84, 95% CI=1.13-13.10, p=0.03); age (OR=1.19, 95% CI=1.09-1.30, p<0.001) was the only other variable that remained a significant predictor of case status. Comparisons with neuropsychological test scores were not carried out due to sample size.

In order to conduct preliminary analyses on the impact of baseline DepE scores on 24-month cognitive change, longitudinal change scores were created for the MMSE and CDR scores (due to missing data and sample size, calculations were not possible with all neuropsychological test data). Of the 90 total TARCC participants with 24-month follow-up data, 65 participants (AD n=41, NC n=49) had all available baseline and 24-month follow-up MMSE and CDR sum of boxes (CDR-SB) scores along with DepE scores and appropriate covariates (age, gender, ethnicity, education; ApoEε4 was not available on sufficient numbers to be included as a covariate). Change scores were calculated as follows: ΔMMSE = baseline MMSE -24mo MMSE and ΔCDR-SB = baseline CDR-SB -24mo CDR-SB. Therefore, a positive ΔMMSE was indicative of decline whereas a negative ΔCDR-SB was indicative of decline. The mean (standard deviation, range) of ΔMMSE and ΔCDR-SB were 2.66 (3.37, 0-16) and -2.23 (3.05, -11-0.5), respectively. Baseline DepE scores were significantly related to both change scores (see Table 3).

## Discussion

The current findings demonstrate that a depressive endophenotype of MCI and AD exists and can be clinically identified using specific items from the GDS-30. Higher scores on this five-item DepE significantly increased risk for diagnosis of MCI in Project FRONTIER and cross-validated by predicting AD in the TARCC. For every one point increase on the scale, the risk of MCI diagnosis increases by a factor of 2 whereas the increased risk for AD was by a factor of nearly 3 (2.8) for every point increase. Even when GDS total score was entered into the predictive model, only the new DepE was a significant predictor of MCI status. ApoEε4 presence did not predict MCI. For AD, when the DepE index was entered into the model neither GDS total scores nor education were significant predictors of AD status. It is noteworthy to point out that the DepE validated in the TARCC cohort despite the substantially lower mean GDS scores when compared to the Project FRONTIER cohort. Therefore, the DepE appears to be identifying a depressive endophenotype of MCI/AD. It is noteworthy that DepE scores were significantly related to neuropsychological test scores consistently across cohorts independent of GDS total scores.

These findings have implications for clinical and research settings. For clinicians, higher scores on this index provide a means of empirically justifying the need for obtaining a formal cognitive evaluation on select depressed elderly patients. Current state-of-the-art diagnoses of AD is obtained through specialty clinic settings incorporating medical examinations, neuropsychological evaluations, clinical blood work, and neuroimaging [52], and this process has been validated against autopsy findings [53]. However, AD is common and under diagnosed in primary care settings [53–55], particularly in the MCI stages [56]. While clinicians are regularly faced with depressed patients who complain of cognitive dysfunction, the decision as to who is or is not in need of formal psychometric assessment has been a judgment call. It is our hope that in the future the DepE may potentially provide a tool for clinicians to justify a referral to an appropriate specialist and our group is conducting work to establish empirical cut-scores from independent sets of patients currently.

From a research standpoint, the current findings point to a depressive endophenotype of MCI and AD. A key purpose for the search for distinct subgroups of individuals at risk AD and MCI is to identify novel treatment and preventative opportunities given that currently available methods have only modest effects. The DepE identifies specific individuals at high risk MCI and AD, which are not identified by total GDS score. Therefore, the DepE may have potential for identifying individuals who would experience cognitive benefit from anti-depressant treatments (pharmaceutical and/or psychotherapy). Specific questions include: can the DepE identify select AD cases that may benefit from anti-depressant treatments for slowing progression of the disease? Can the DepE be utilized to select particular MCI patients for anti-depressant trials aimed at slowing progression to AD? Can the DepE index identify cognitively normal elders at risk for MCI and/or AD and will anti-depressant treatments slow such development? Can the DepE index identify MCI cases more likely to “revert” to normal rather than progress to AD? Prior animal model work suggests potential for anti-depressant treatments on cognition/AD [57,58] can have cognitive benefit; however, implementation of this into humans has not been successful [59]. The current findings may offer a way of translating that work into clinical trials.

There are weaknesses to the current study. The sample size across cohorts was relatively modest; however, the effect size was sufficiently large to be detected. The number of MCI cases available for analysis from the TARCC database was limited as were the number of cases available for longitudinal analysis. Additionally the available cases were predominately Mexican-Americans and non-Hispanic whites which limits the generalizability of the findings. The TARCC cohort monitors over 1500 participants annually and future work will collect additional item-level GDS scores for calculation of the DepE for further cross-validation. Data related to previously diagnosed mental illness and antidepressant use was not available for all participants; however psychiatric illness was an exclusionary criteria for the TARC sample. Taken together, the current findings offer a significant advancement to the extant literature. The DepE appears to identify a depressive endophenotype of MCI and AD. Future research should cross-validate the endophenotype among additional cohorts, investigate the biology of the link between the index and MCI/AD risk, identify specific guidelines for clinical implementation, and determine if the index is useful for clinical trials aimed at treating and/or preventing MCI and AD.
